# Myringotomy: Incision of the Tympanic Membrane

**Published:** 1907-05-11

**Authors:** 


### Otology.

### MYRINGOTOMY: INCISION OF THE TYMPANIC MEMBRANE.

The precise care with which the tympanic membrane is incised undoubtedly influences the course of acute otitis media. The technique of the operation is as follows: A general anæsthetic should be given, for though the operation can be performed without this, an anæsthetic is certainly of the greatest assistance to the operator in making a free incision. Local anæsthesia is not easily attained for operations upon the ear. The following instruments are required: 1. An aural speculum, which should be selected to fit the meatus and to give the best view of the membrane. 2. A head-lamp or mirror, which must give a good light. 3. A pair of prehensile aural forceps for removal of adherent *débris*, etc. 4. A fine pair of aural forceps for carrying small wool swabs. These latter forceps should have blades about half or one-third the thickness of those usually sold by instrument makers. The spring should be weak. 5. A syringe, all-metal, including the plunger, with capacity of 2 to 4 ounces. If this is not available a glass syringe, in good working order, will answer the purpose satisfactorily. 6. A myringotome; this should be a finely pointed, keen edged knife, with short blade on long handle. The shaft should be straight.

It is essential that all meatal *débris* should be removed. This is usually accomplished by syringing. After syringing the meatus must be dried with sterilised wool.

With the exception of the myringotome, the instruments should be boiled before use. The myringotome should be sterilised with pure carbolic acid. Its blade should not be passed through a flame, for this spoils the temper of the steel. (This instrument should be treated with care, especially to protect its point from injury, for a good myringotome is a fine tool, and easily ruined.)

The patient having been anæsthetised, the speculum is inserted and light thrown upon the membrane. The point of the myringotome should be directed towards the lower part of the membrane in view with the cutting edge upwards. There should be a sharp stabbing action in perforating the drum, *but the point of the knife is not withdrawn until the incision is completed across the drum.* Whether a curved or straight incision is made, is of less importance than the freedom with which it is made. Nor is it material whether the incision is parallel with the handle of the malleus or not, so long as the malleus itself is not disturbed. The part of the membrane which bulges is the part which must be incised. This is usually posterior to the malleus. The incision is made by a lever-like movement of the knife, the handle of which rests upon the meatal wall as a fulcrum. (The method above described is to be preferred to that of puncture with a double-edge blade, which, in the writer's opinion, does not ensure the drainage which it is intended to obtain.) For purposes of bacteriological examination the fluid in the middle ear should be withdrawn with a sterile pipette, or the blade of the knife may be transferred to the culture media forthwith.

After the drum has been incised, the meatus should be gently syringed with hot, weak, alkaline lotion, then dried with sterilised absorbent wool, and lastly loosely packed with a narrow strip ($\frac{3}{4}$ inch) of ribbon gauze, which should be in contact with the incision in the membrane. If there is much oozing a light outside dressing should be applied, and the whole dressing changed in six or twelve hours, the meatus being gently syringed with weak alkaline and boric lotion. In a few days the membrane should be healed.

